# Primoridal germ cell specification: a context-dependent cellular differentiation event

**DOI:** 10.1098/rstb.2013.0543

**Published:** 2014-12-05

**Authors:** Ufuk Günesdogan, Erna Magnúsdóttir, M. Azim Surani

**Affiliations:** 1 Wellcome Trust/Cancer Research UK Gurdon Institute, University of Cambridge, Tennis Court Road, Cambridge CB2 1QN, UK; 2 Department of Physiology, Development and Neuroscience, University of Cambridge, Downing St., Cambridge CB2 3DY, UK; 3 Wellcome Trust Medical Research Council Stem Cell Institute, University of Cambridge, Tennis Court Road, Cambridge CB2 1QR, UK; 4 Department of Biochemistry and Molecular Biology, BioMedical Center, University of Iceland, 101 Reykjavík, Iceland

**Keywords:** primordial germ cells, specification, epigenetic reprogramming, developmental competence, enhancer

## Abstract

During embryonic development, the foundation of the germline is laid by the specification of primordial germ cells (PGCs) from the postimplantation epiblast via bone morphogenetic protein (BMP) and WNT signalling. While the majority of epiblast cells undergo differentiation towards somatic cell lineages, PGCs initiate a unique cellular programme driven by the cooperation of the transcription factors BLIMP1, PRDM14 and AP2γ. These factors synergistically suppress the ongoing somatic differentiation and drive the re-expression of pluripotency and germ cell-specific genes accompanied by global epigenetic changes. However, an unresolved question is how postimplantation epiblast cells acquire the developmental competence for the PGC fate downstream of BMP/WNT signalling. One emerging concept is that transcriptional enhancers might play a central role in the establishment of developmental competence and the execution of cell fate determination. Here, we discuss recent advances on the specification and reprogramming of PGCs thereby highlighting the concept of enhancer function.

## 1. Introduction

Primordial germ cells (PGCs), the precursors of the gametes, represent a cell lineage with unique properties. They are unipotent and differentiate into sperm and oocytes, depending on the sex of the organism. Fertilization of the oocyte by sperm generates the totipotent zygote, which is the founder cell of all lineages of an organism. In addition, PGCs in culture can give rise to pluripotent embryonic germ cells [1,2], which closely resemble embryonic stem (ES) cells [3]. Thus, the PGC lineage differentiates and at the same time acquires the capacity to become totipotent.

**Figure 1.**
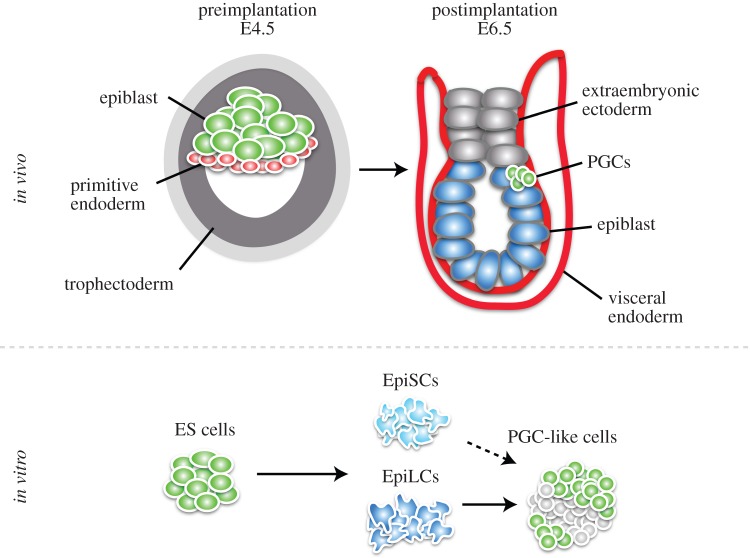
Embryonic origin of PGCs *in vivo* and PGC derivation *in vitro*. The preimplantation blastocyst at E4.5 consists of the embryonic lineage, the epiblast, and two extraembryonic lineages, primitive endoderm and trophectoderm. After implantation at E6.5, signalling from the extraembryonic ectoderm as well as from the visceral endoderm induces a few cells of the proximal epiblast to become PGCs. *In vitro*, ES cells, which are derived from the preimplantation epiblast, can be differentiated into epiblast-like cells (EpiLCs) with Activin A and basic fibroblast growth factor. EpiLCs, in turn, respond to BMP4 to give rise to functional PGC-like cells. Epiblast stem cells (EpiSCs), which are traditionally derived from the postimplantation epiblast, also give rise to PGC-like cells, but at a low frequency. (Online version in colour.)

Mammals specify their PGCs in response to instructive signalling during embryonic development. In mice, the blastocyst differentiates into epiblast, trophectoderm and primitive endoderm (figure 1). The epiblast develops into the embryo proper, whereas the other two lineages give rise to extraembryonic tissues. The latter not only develop into essential structures such as the placenta to support the development of the embryo but also act as signalling sources to allocate lineages to the epiblast cells. Bone morphogenetic protein (BMP) signalling at embryonic day (E) 6.25 after implantation from the extraembryonic tissue to the proximal epiblast results in the specification of the germ cell lineage, by assigning a few epiblast cells to become PGCs [4,5]. While the remaining epiblast cells initiate differentiation towards somatic cell lineages, nascent PGCs reverse this programme by switching on the expression of a transcriptional network including BLIMP1, PRDM14 and AP2γ [6–8]. These factors drive transcriptional changes and epigenetic remodelling in part by inducing the re-expression of pluripotency genes and repressing the DNA methylation machinery, accompanied by genome-wide DNA demethylation, X chromosome reactivation, erasure of imprints and dynamic changes in histone modification signatures [9–14]. During this reprogramming event, PGCs proliferate and migrate towards the genital ridges, which they colonize by E10.5. Female PGCs enter meiosis at approximately E12.5 to produce oocytes, and male PGCs induce a mitotic arrest at approximately E13.5 before they undergo spermatogenesis.

At the time of PGC specification, postimplantation epiblast cells exhibit a state of primed pluripotency; they express some pluripotency-associated genes together with lineage commitment genes. Moreover, these cells show signs of differentiation such as DNA methylation and an inactive X chromosome (in female embryos) [15–19]. Although they are able to differentiate into all embryonic cell lineages, they do not contribute to chimaeras when injected into blastocysts. So far, all attempts to directly differentiate PGCs *in vitro* from pluripotent cell types that are distinct from the postimplantation epiblast worked at extremely low efficiency or have failed altogether. The question of how postimplantation epiblast cells gain the developmental potential to become PGCs remains unclear.

One emerging concept is that enhancer elements play a pivotal role in defining or at least contributing to the establishment of developmental potential. Enhancer elements can be defined as *cis*- (and in a few cases *trans-*) acting DNA sequences that contain multiple transcription factor binding sites, which activate transcription of associated genes independent of their location, orientation or distance relative to gene promoters. Intriguingly, enhancers exhibit cell type-specific patterns of combinatorial histone modifications, which correlate with their activity. Inactive enhancers also carry a specific epigenetic signature in progenitor cells before they become activated upon signalling, which led to the hypothesis that enhancers exist in a ‘poised’ state prior to their activation. Thus, the epigenetic set-up of enhancers might also be a prerequisite for the gradual process of lineage commitment and differentiation in the context of PGC specification from postimplantation epiblast cells.

In this review, we will discuss the specification and reprogramming of PGCs and, in particular, the principles of enhancer function with examples from different systems.

## 2. Induction of the primordial germ cell fate

**Figure 2.**
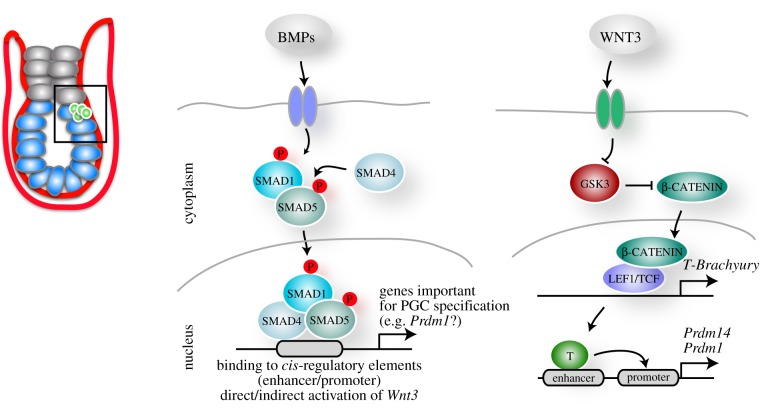
The role of BMP and WNT signalling in PGC specification. BMP4 emanating from the extraembryonic ectoderm and BMP2 from the visceral endoderm induce the phosphorylation of SMAD1 and SMAD5, which form a complex with SMAD4. This complex translocates into the nucleus and presumably binds enhancers and promoters of genes that are required to establish PGC fate. Moreover, BMP signalling results in the direct or indirect activation of WNT3, which is also required to induce the PGC fate. Most likely, WNT3 signalling results in the repression of GSK3, which in turn causes the stabilization of β-CATENIN. β-CATENIN translocates into the nucleus and together with LEF1/TCF activates the expression of *T-Brachyury*. T-BRACHYURY binds to enhancers which are in close proximity to the promoters of *Prdm1* (encoding BLIMP1) and *Prdm14*. (Online version in colour.)

The germ cell lineage is formed via distinct modes in the animal kingdom. Some species such as *Caenorhabditis elegans* or *Xenopus laevis* rely on maternally inherited determinants, which segregate asymmetrically to the prospective PGCs [20]. Other species, including mammals, specify their PGCs in response to signalling during embryonic development. Indeed, some invertebrates such as the cricket *Gryllus bimaculatus* also induce PGCs through BMP signalling [21,22]. In mice, BMP signalling is required for mesoderm development and PGC specification. BMP4 and BMP8b secreted from the extraembryonic ectoderm at E6.0 towards the proximal epiblast induce a few cells in the posterior of the embryo to become PGCs (figure 1) [4,5,23]. BMP4 is sufficient to induce PGCs, whereas BMP8b controls the development of the visceral endoderm, which is a source of inhibitory signals including LEFTY1 and CER1 for BMP4 [24]. BMP2 is expressed in the visceral endoderm, which surrounds the epiblast, and presumably augments the BMP4 signal in the posterior of the embryo [24,25]. The BMP4 signal acts through a receptor complex including BMP receptor type II and ALK3/6, which results in SMAD1/5 phosphorylation (figure 2). SMAD1/5 form a complex with SMAD4 and translocate to the nucleus to control target gene expression. The importance of this pathway is demonstrated by studies with mutations in *Bmp4*, *Bmp8b*, *Smad1* and *Smad5* as they show impaired PGC development [5,26,27]. The exact target genes of the BMP pathway in the prospective PGCs remain to be identified. However, BMPs trigger the activation of a transcriptional network with the key regulators BLIMP1 and PRDM14, while AP2γ is induced by BLIMP1 [28,29]. This is followed by the re-expression of pluripotency genes such as *Nanog* and *Sox2*.

BMP signalling is also required in a dose-dependent manner for maintaining pluripotency of ES cells [30]. ChIP-seq (chromatin immunoprecipitation followed by massively parallel sequencing) studies for a number of transcription factors in ES cells revealed that the BMP target SMAD1 together with OCT4, SOX2, NANOG and STAT3 co-bind loci that are mainly in intergenic regions, where enhancers reside, to drive ES-specific gene expression [31]. Some of these factors also promote the recruitment of p300, a transcriptional co-activator and histone acetyltransferase known to bind to enhancers [32]. Thus, SMAD1, the downstream mediator of the BMP pathway, cooperates with additional transcriptional factors to bind to enhancers in a combinatorial way to execute the ES cell programme. Of note, the binding of these factors is interdependent, as the knockdown of individual proteins reduces the binding affinity of their partners [31]. This provides one explanation for the basic question of how the same transcription factors in different combinations regulate distinct cellular identities. Accordingly, a study on the haematopoietic lineage shows that SMAD1 together with TCF712, a transcription factor mediating WNT signalling, co-occupies enhancers in haematopoietic stem cells [33]. Upon erythropoiesis, the binding of these factors gets directed to a narrower set of enhancers, which are occupied by the lineage-specific GATA factors, refining the transcriptional output. Thus, SMAD1 binds specific subsets of enhancers in a context-dependent manner and it will be of particular interest to identify the SMAD1/5 targets and its partners acting to initiate the PGC programme.

A second signalling pathway that is required for the induction of PGCs is the WNT pathway mediated by WNT3/β-CATENIN (figure 2) [34]. *Wnt3* is expressed at first in the posterior visceral endoderm at about E5.5, and then additionally in the posterior epiblast at approximately E5.75 [35], which precedes the time of PGC specification. A mutation in *Wnt3* results in defects in gastrulation and primitive streak formation [36], and mutant epiblasts fail to give rise to PGCs [24]. One of the downstream targets of WNT3 is the *T* gene, which encodes the T-box transcription factor T*-*BRACHYURY, and its role in mesoderm formation has been extensively described [37]. A recent study revealed that T*-*BRACHYURY expression is also essential and sufficient for PGC specification [34]. T-BRACHYURY binds to putative enhancers that are in close proximity to *Prdm1* and *Prdm14*, but further studies are required to determine whether this binding directly activates the expression of these genes. Although many functionally characterized enhancers are in close proximity to their target promoters, a few examples exist of long-range interactions such as the enhancer of *Sonic hedgehog*, which is located approximately 1 Mb away in an intron of the *Lmbr1* gene [38]. Indeed, new emerging technologies such as chromosome conformation capture (3C) in combination with next generation sequencing (4C, 5C, Hi-C) or ChIA-PET (chromatin interaction analysis by paired-end tag sequencing) revealed that the majority of enhancers do not target the nearest promoter [39,40].

Another open question is how the WNT pathway induces two distinct lineages from the same set of progenitor epiblast cells, the mesodermal and PGC lineage. There appears to be an intricate balance between the timing of signalling events and the precise order in which they act to induce target gene expression. For example, priming of cultured epiblast cells (see below) with WNT prior to BMP exposure inhibits the induction of the PGC fate [34]. The activation of BMP signalling thus seems to be required in prospective PGCs to provide competence for WNT-mediated induction of the PGC fate through T-BRACHYURY.

## 3. Developmental competence to become primordial germ cells

During embryonic development, only a few cells of the proximal epiblast are destined to become PGCs. However, early experiments suggested that not only the proximal epiblast cells exhibit the developmental competence for PGC fate acquisition. Distal epiblast cells could also respond to BMP signalling and adopt the PGC fate when transplanted posteriorly to the proximal epiblast adjacent to the BMP signal emanating from the extraembryonic ectoderm [41,42]. More recently, it was shown that the majority of cells of the epiblast, separated from the visceral endoderm and/or extraembryonic ectoderm, can adopt the PGC fate upon addition of BMP4 to the culture medium [24], which shows that most postimplantation epiblast cells are generally competent to become PGCs. However, this competence exists only for a short duration during development in the epiblast from E5.5 to E6.5 embryos. In part this is due to inhibitory signals from the visceral endoderm, including CER1, LEFTY1 and DKK1 that inhibit posteriorization and consequently restrict the specification of the PGC fate to the posterior proximal epiblast. Accordingly, *Smad2^−/–^* embryos that lack the BMP inhibitor CER1 fail to restrict PGC induction [24].

Cells from the postimplantation epiblast can give rise to self-renewing and pluripotent epiblast stem cells (EpiSCs) in culture under appropriate culture conditions. Traditionally, this cell type was derived from the epiblast of E5.5–E6.5 embryos [43,44], but more recently EpiSCs were also successfully derived from E3.5 preimplantation embryos and from E6.5 to E8.0 embryos [45,46]. EpiSCs in some ways resemble *in vivo* postimplantation epiblast cells as they also show higher expression of lineage-determining genes such as *Fgf5* and *Lefty1* as well as lower expression of pluripotency genes as compared with ES cells and exhibit an inactivate X chromosome in female cells. EpiSCs retain the capability to become PGC-like cells *in vitro* [47]. They express BMP4 and continuously specify PGC-like cells under self-renewing conditions, but at very low frequency. It was recently shown that EpiSCs are very similar to the ectoderm of the late gastrula stage irrespective of the developmental stages they were derived from [48]. Thus, EpiSCs reflect a later developmental stage compared to that when PGCs are specified *in vivo*, which could explain the low efficiency of PGC derivation *in vitro*.

The appropriate conditions to efficiently derive PGCs directly from ES cells *in vitro* are not yet identified. However, when ES cells are injected into blastocysts, they can contribute to all embryonic lineages including the germline and under certain culture conditions even give rise to extraembryonic lineages [49,50]. Thus, it could be that ES cells must transit to a primed epiblast-like state first as *in vivo*, before they gain the competence to efficiently give rise to PGCs *in vitro*. Indeed, such a two-step differentiation method was recently developed (figure 1) [51,52]. ES cells were differentiated into epiblast-like cells (EpiLCs) using Activin A and basic fibroblast growth factor (FGF), the same cytokines used to culture EpiSCs under self-renewing conditions. After 2 days of differentiation, EpiLCs exhibit a similar transcriptional profile to postimplantation epiblasts from E5.75 embryos [51], which corresponds to the developmental stage, when they become competent to induce the PGC fate. EpiLCs then respond to BMP4 by giving rise to functional PGC-like cells at high frequency.

WNT3 signalling is required for the competence of the epiblast cells to induce germline determinants downstream of BMP4 and subsequently give rise to PGCs [24,34]. PGC-like cells can be derived from *Wnt3* mutant EpiLCs via overexpression of T-BRACHYURY, which suggests that WNT3 induces the PGC fate directly, where T-BRACHYURY is activated by β-CATENIN downstream of WNT3 (figure 2) [34]. Interestingly, in the absence of BMP signalling, T-BRACHYURY fails to induce BLIMP1 and PRDM14, indicating that BMP4 is required for the competence of epiblast cells to respond to WNT signalling to induce the PGC fate. This collaboration between WNT and BMP signalling indicates a more complex scenario than previously anticipated.

**Figure 3.**
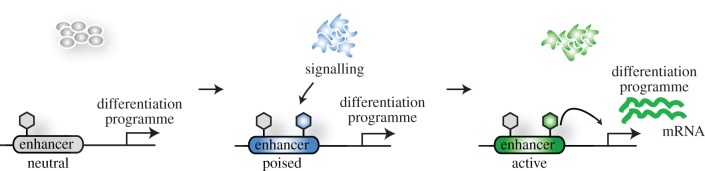
Model of the enhancer set-up during differentiation. During differentiation, a set of neutral enhancers changes its epigenetic profile and become poised for activation. These enhancers can be engaged by factors downstream of signalling pathways, activating them to induce the transcriptional programme of a specific differentiation programme. (Online version in colour.)

One concept that needs functional testing is whether enhancers are set up via a cell type-specific histone modification signature in order to prime them for activation, thereby contributing to the developmental potential of a cell type (figure 3). Enhancer usage is a dynamic process during differentiation as exemplified by the gene *Pou5f1* (encoding OCT4), which is alternately under the control of its distal and proximal enhancers depending on the developmental context [53]. The preimplantation epiblast, ES cells and PGCs engage the distal enhancer for *Pou5f1* expression, whereas the postimplantation epiblast and EpiSCs make use of the proximal enhancer [53,54]. This switch in enhancer utilization for *Pou5f1* has been exploited extensively for the experimental manipulation of PGCs, and it is interesting to speculate that it is a marker of global alterations of enhancer engagement as PGC specification commences. Moreover, recent studies show that enhancers exhibit a defined set of histone modifications during differentiation. For example, H3K4me1 and H3K27ac mark an active set of enhancers in human ES cells, whereas primed or ‘poised’ enhancers are marked by H3K4me1 and H3K27me3, which are associated with developmental genes [55]. Upon differentiation of human ES cells into neuroectodermal spheres, a subset of poised enhancers become activated and change their epigenetic profile by losing H3K27me3 but gaining H3K27ac. There is accumulating evidence that these histone modifications are indeed causative for enhancer activity and not just a consequence [56]. For example, the TALEN system was used to recruit the histone demethylase LSD1 to target enhancers to remove H3K4 methylation, which resulted in a decrease in their activity [57]. In addition, the histone methyltransferase MLL4 was shown to catalyse the deposition of H3K4me1 at enhancers in a tissue-specific manner during adipogenesis and myogenesis [58]. Thus, the set-up of the enhancer landscape might potentially contribute to the establishment of developmental competence during the initiation of differentiation from the inner cell mass of the blastocyst towards the postimplantation epiblast prior to PGC specification.

## 4. The primoridal germ cell-specific transcriptional network

Upon BMP signalling, prospective PGCs sequentially activate the expression of the three key transcription factors BLIMP1, PRDM14, followed by AP2γ. Although these factors in combination set up the initial condition required for reprogramming of PGCs, each one of them is also involved in other processes and expressed in distinct tissues: BLIMP1 has been well characterized in the functional differentiation of B and T lymphocytes and other haematopoietic lineages as well as in the epidermis, with various different functions during development [59–62]; PRDM14 is required for maintaining naive pluripotency in ES cells by repressing FGF signalling and DNA methylation [63,64]; AP2γ is involved in the development of extraembryonic tissues among others [65]. However, their combined presence results in the emergent properties of nascent PGCs when expressed at the correct developmental time point [28,29].

**Figure 4.**
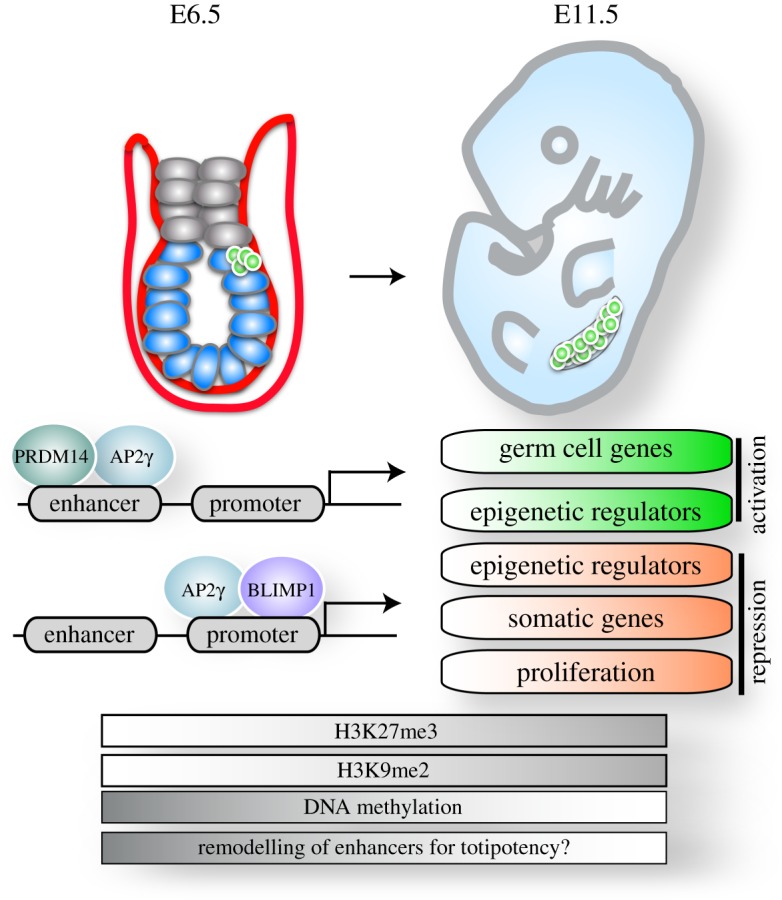
Epigenetic reprogramming of PGCs. Upon specification of PGCs at E6.5, a PGC-specific transcriptional network including BLIMP1, PRDM14 and AP2γ is activated. PRDM14 binds predominantly to enhancers, whereas BLIMP1 occupies mainly promoters. AP2γ shows bimodal enrichment and binds to both distal regulatory elements as well as promoters. These transcription factors synergistically repress somatic and proliferation genes and the expression of some epigenetic regulators. Further, they activate the expression of another set of epigenetic regulators as well as germ cell genes. These transcriptional changes are accompanied by epigenetic remodelling: H3K9me2 is progressively lost, H3K27me3 is enriched and DNA is globally demethylated. These epigenetic changes could potentially contribute to the remodelling of the enhancer landscape towards the capacity of totipotency. (Online version in colour.)

In prospective PGCs, the three factors execute changes in different branches of the gene expression programme (figure 4) [28]. Firstly, they suppress the ongoing somatic differentiation through repressing the expression of somatic regulators. Secondly, they repress genes required for DNA methylation, thus enabling DNA demethylation. Furthermore, they slow down cell proliferation by repressing genes essential for cell cycle progression and cell growth. Simultaneously, they induce PGC-specific gene expression by driving the reinstatement of a subset of the pluripotency network concurrent with the induction of germ cell genes such as *Rhox* genes, *Nanos3* and *Dnd1*. In addition, these factors differentially regulate several histone modifiers such as *Kdm6b*, *Kdm3a* and *Kdm4b* that act to alter the chromatin state of nascent PGCs. Remarkably, the three factors can completely substitute for cytokines and are sufficient for the induction of the PGC fate from EpiLCs in culture [28,29]. Individual factors were able to also induce the PGC fate but at decreased efficiencies, and the resulting PGCs always induced the expression of the remaining factors [29]. In fact, genetic experiments have shown that all three factors are essential for PGC development *in vivo* [6–8], which most likely is also the case *in vitro*.

Interestingly, BLIMP1, PRDM14 and AP2γ have distinct binding patterns relative to promoters. BLIMP1 is mainly enriched at transcriptional start sites, whereas PRDM14 is predominantly bound to enhancers (figure 4) [28,66]. These two factors are thus likely to collaborate on the regulation of gene expression through distinct mechanisms. AP2γ, on the other hand, appears to bridge this relationship by showing bimodal enrichment, both on distal regulatory elements as well as on promoters. This binding pattern exhibited by these three essential factors underscores the importance of regulating gene expression both at the level of enhancers as well as promoters when driving cellular commitment. In fact, BLIMP1 is mainly responsible for direct gene repression, with some notable exceptions, and collaborates in repressing somatic genes with the other two factors. It thus seems likely that the most efficient way of repressing gene expression is by directly repressing promoter activity, whereas the activation of robust gene expression *de novo* is likely to require enhancer engagement and activation.

Interestingly, OCT4 along with SOX2 and KLF4 has been shown to exhibit pioneer factor function during somatic cell reprogramming, where they co-bind a large set of DNAse-I inaccessible sites that are devoid of histone modifications [67]. Pioneer transcription factors have the ability to bind to DNA sequences recruiting cofactors, causing chromatin remodelling and rendering the site accessible to other factors [68,69]. The binding of OCT4, SOX2 and KLF4 is a prerequisite for the binding of Myc, which in turn enhanced the binding of the other three, showing that indeed OCT4, SOX2 and KLF4 act as pioneer factors to initiate reprogramming and access enhancers that have been rendered inactive by heterochromatization, preceding downstream changes in transcriptional activity [67]. The action of the three PGC specifying factors BLIMP1, PRDM14 and AP2γ is highly context dependent, suggesting that they are unlikely to function as pioneer factors. OCT4 is present both in ES cells and in the PGC competent state of the primed pluripotent epiblast cells and thus is also not likely to act as a pioneer factor allowing the subsequent binding of the three factors to their PGC target sites. Whether there exist other factors that can act as pioneer factors for priming the action of the tripartite network for PGC induction remains to be addressed.

## 5. Epigenetic reprogramming of primordial germ cells

The transcriptional changes that are initiated after PGC specification are connected to extensive and dynamic epigenetic reprogramming including global DNA demethylation and changes in the profile of histone modifications (figure 4) [9–14]. Generally, DNA methylation at cytosines (5-methylcytosine; 5mC) regulates chromatin structure as well as gene transcription to stabilize a cell lineage-specific gene expression profile. During embryonic development, its genome wide erasure and reinstatement correlates with gain of developmental competence and lineage determination, respectively [70]. Shortly after PGC specification at E8.5–E12.5, the global erasure of DNA methylation resets the epigenome of PGCs including genomic imprints. The latter are subsequently re-established gender specifically and passed on to the next generation, giving rise to the monoallelic expression of imprinted genes in a parent-of-origin-specific manner [71]. Furthermore, methylation-dependent genes that are expressed specifically in the germline, such as *Dazl*, *Ddx4* and *Rhox* genes, become activated [72–74]. It is tempting to speculate that global DNA demethylation in PGCs contributes to a switch in the enhancer usage to establish the germline-specific gene expression programme. Several studies suggest a functional relationship of enhancers and DNA methylation, as enhancer activity inversely correlates with DNA methylation. Differentially methylated regions identified via the generation of DNA methylation maps using a number of adult mouse tissues were predominantly located at putative enhancer elements [75]. Furthermore, active enhancers marked with H3K4me1 and H3K27ac in ES cells show enrichment of 5-hydroxymethylation (5-hydroxymethylcytosine; 5hmC [76–78], an intermediate in the DNA demethylation pathway). Intriguingly, global DNA demethylation in PGCs is driven in part by TET1 and TET2, which catalyse the conversion of 5mC to 5hmC, which is then removed via DNA replication-coupled dilution [14]. Thus, the erasure of DNA methylation could potentially open up the opportunity to activate a new set of enhancers.

Of note, at the time of global DNA demethylation, PRMT5, an arginine methyltransferase, translocates from the cytoplasm to the nucleus specifically in PGCs [79]. PRMT5 catalyses the methylation of H2A/H4R3me2, but its functional importance in PGCs remains to be investigated.

DNA demethylation in PGCs takes place in concert with dynamic global changes in histone modifications. There is a progressive loss of H3K9me2 at E7.75–E8.75 [10,11,13], which can be due to the repression of the methyltransferase GLP. It is unlikely that the erasure of H3K9me2 is due to a passive dilution during DNA replication, since the PGC-specific transcriptional network represses proliferation factors [28], and PGCs undergo a G2 arrest during this developmental period [11]. Thus, an induction of demethylases and/or the replacement of the entire histone H3 molecule by its non-canonical counterpart H3.3 are presumably involved in this event. In parallel, from E8.25 until E9.5, H3K27me3, a mark that is deposited by polycomb repressive complex 2 (PRC2), progressively increases in PGCs and changes in distribution from being concentrated on the inactive X chromosome in female embryos to having a more even distribution throughout the nucleus [10,11,13]. The functional implications of these changes are unclear because both histone marks are associated with transcriptional repression and it would be interesting to see which loci lose or gain these marks over time. Notably, a recent study analysed local enrichment of histone modifications via low cell number chromatin immunoprecipitation using PGCs at different developmental time points (E11.5, E13.5 and E15.5), identifying a germ cell-specific set of active enhancers [80]. Moreover, H3K27me3 seems to be enriched not only at developmental genes but also at genes involved in immune system activation, which were germ cell specific when compared to other cell types including ES cells. Although further studies are required to clarify the underlying mechanisms and the functional implications of epigenetic reprogramming during PGC development, it seems that PGCs acquire a unique epigenetic set-up that most likely shapes the enhancer landscape by activating, poising or inactivating them in preparation for totipotency.

## 6. Concluding remarks

With the advent of a novel culture system for PGC induction, molecular events driving the specification and differentiation of this unique cell lineage are being uncovered at an ever increasing pace. Systematic investigations have revealed a precise order of signalling events required for the arrest of mesodermal induction simultaneous to the retention of underlying pluripotency and the push towards the unipotent state of developing PGCs that undergo an epigenetic re-setting event prior to meiosis or spermatogenesis. Although these events have been extensively studied and well described, we are still at the beginning of understanding the underlying mechanisms and their functional implications. The fact that even the powerful combination of the three transcription factors BLIMP1, PRDM14 and AP2γ is not sufficient for inducing this cell fate until the correct developmental window is reached in the pluripotent postimplantation epiblast cells or their *in vitro* counterpart has opened up further questions of developmental plasticity and competence.

We have here described molecular events shaping the process of PGC specification and reprogramming with a specific perspective on the role of transcriptional enhancers in cell fate specification. The concept that the epigenetic state of enhancers defines or at least contributes to developmental competence remains to be elucidated and functional studies are required to identify the true nature of their role in development.
